# Microscopic-Observation Drug-Susceptibility Assay for the Diagnosis of Drug-Resistant Tuberculosis in Harare, Zimbabwe

**DOI:** 10.1371/journal.pone.0055872

**Published:** 2013-02-11

**Authors:** Beauty Makamure, Jesca Mhaka, Salome Makumbirofa, Reggie Mutetwa, Lucy Mupfumi, Peter Mason, John Z. Metcalfe

**Affiliations:** 1 Biomedical Research and Training Institute, Harare, Zimbabwe; 2 University of Zimbabwe College of Health Sciences, Harare, Zimbabwe; 3 Francis J. Curry International Tuberculosis Center, Division of Pulmonary and Critical Care Medicine, San Francisco General Hospital, University of California San Francisco, San Francisco, California, United States of America; National Institute of Allergy and Infectious Diseases, United States of America

## Abstract

**Introduction:**

Limited data exist on use of the microscopic-observation drug-susceptibility (MODS) assay among persons suspected of MDR-TB living in high HIV-prevalence settings.

**Methods:**

We retrospectively reviewed available clinical and drug susceptibility data for drug-resistant TB suspects referred for culture and drug-susceptibility testing between April 1, 2011 and March 1, 2012. The diagnostic accuracy of MODS was estimated against a reference standard including Löwenstein-Jensen (LJ) media and manual liquid (BACTEC MGIT) culture. The accuracy of MODS drug-susceptibility testing (DST) was assessed against a reference standard absolute concentration method.

**Results:**

One hundred thirty-eight sputum samples were collected from 99 drug-resistant TB suspects; in addition, six previously cultured MDR isolates were included for assessment of DST accuracy. Among persons with known HIV infection status, 39/59 (66%) were HIV-infected. Eighty-six percent of patients had a history of prior TB treatment, and 80% of individuals were on antituberculous treatment at the time of sample collection. *M. tuberculosis* was identified by reference standard culture among 34/98 (35%) MDR-TB suspects. Overall MODS sensitivity for *M. tuberculosis* detection was 85% (95% CI, 69–95%) and specificity was 93% (95% CI, 84–98%); diagnostic accuracy did not significantly differ by HIV infection status. Median time to positivity was significantly shorter for MODS (7 days; IQR 7–15 days) than MGIT (12 days; IQR 6–16 days) or LJ (28 days; IQR 21–35 days; p<0.001). Of 33 specimens with concurrent DST results, sensitivity of the MODS assay for detection of resistance to isoniazid, rifampin, and MDR-TB was 88% (95% CI, 68–97%), 96% (95% CI, 79–100%), and 91% (95% CI, 72–99%), respectively; specificity was 89% (95% CI, 52–100%), 89% (95% CI, 52–100%), and 90% (95% CI, 56–100%), respectively.

**Conclusion:**

In a high HIV-prevalence setting, MODS diagnosed TB and drug-resistant TB with high sensitivity and shorter turnaround time compared with standard culture and DST methods.

## Background

The World Health Organization (WHO) estimates that 310,000 cases of multidrug-resistant tuberculosis (MDR-TB) occurred in 2011, although less than one in five cases were detected. [1] The emergence of MDR/XDR-TB in the southern Africa region in particular has been associated with high mortality [2], [3] and may be substantially underestimated [4]–[6].

Accurate, timely, and affordable drug susceptibility testing (DST) for surveillance and patient management in high burden countries is urgently needed. Fewer than one-half of the 46 countries in the WHO African Region have provided representative data concerning the prevalence of drug resistance among *M. tuberculosis* strains, and only ten have reported such data since 2000. [5] The microscopic-observation drug-susceptibility (MODS) assay is an accurate, inexpensive, liquid culture-based diagnostic test that has been endorsed by the WHO for rapid screening of patients suspected of having MDR-TB. [7], [8] Despite rationale for expanded use of the MODS assay in high HIV-prevalence regions, [9] diagnostic accuracy data among HIV-infected TB and MDR-TB suspects remain limited [10], [11].

In order to examine the diagnostic accuracy of the MODS assay for *M. tuberculosis* detection and direct DST among MDR-TB suspects in a high HIV-prevalence region, we compared test results against a solid and liquid culture reference standard.

## Methods

### Study Population

We retrospectively reviewed available clinical and drug susceptibility data for drug-resistant TB suspects referred for culture and drug-susceptibility testing between April 1, 2011 and March 1, 2012. Drug-resistant tuberculosis suspects were defined by either (1) history of prior treatment (>1 month, classified according to World Health Organization criteria [12]) or (2) contact with an individual with known or suspected drug-resistant TB. Samples were obtained from patients suspected of having drug-resistant TB by community clinicians, and from participants of ongoing clinical studies. In addition, six previously cultured MDR isolates were included for assessment of DST accuracy. Participants of ongoing clinical studies provided written informed consent, and ethical approval was obtained from the Medical Research Council of Zimbabwe, the Institutional Review Board of the Biomedical Research and Training Institute, and the UCSF Human Research Protection Program. De-identified data from patients not participating in ongoing clinical studies and seen in the course of routine medical practice did not meet the definition for human subjects and were exempt from ethics review.

### Laboratory Methods

The Biomedical Research and Training Institute (BRTI) Tuberculosis Laboratory within the National Microbiology Reference Laboratory (NMRL) is a center for Trials of Excellence in Southern Africa (TESA). BRTI collaborates with the Ministry of Health and Child Welfare (MOHCW) in laboratory capacity building and regularly undergoes External Quality Assurance (EQA) of DST for first-line anti-TB drugs. The most recent Centre for American Pathologists (CAP) assessment in 2012 demonstrated 100% agreement for isoniazid, rifampicin, ethambutol, and streptomycin resistance testing.

Sputum specimens were transported to the BRTI Tuberculosis Laboratory for standard culture, DST, and MODS testing within 48 hours of collection. Each sample was divided into two aliquots: the first aliquot underwent sputum AFB smear examination, decontamination, culture, and DST according to published guidelines, [13] and the second aliquot underwent MODS testing. Laboratory technicians interpreting index test results were blinded as to reference standard test results, and vice versa.

In preparation for reference standard culture and DST, the first aliquot was digested using the 4% sodium hydroxide method. The resuspended sediment was used to make a concentrated smear and inoculated onto Löwenstein-Jensen (LJ) media and in BBL™ MGIT™ Mycobacterial Growth Indicator Tubes (Becton Dickinson, Sparks, MD). MGIT broth tubes were continuously monitored for 40 days for *M. tuberculosis* growth by use of a manual MGIT reader. [14] Ziehl-Neelsen staining was used to confirm growth of Mycobacteria in all test positive tubes. MGIT cultures that had a mixture of mycobacteria and other bacterial contamination from 21 to 40 days were again decontaminated and re-cultured. All positive cultures by MGIT were identified as *M. tuberculosis* complex by MPT64 antigen detection, [15] and by growth at different temperatures if antigen detection was negative. LJ media were monitored for *M. tuberculosis* growth weekly for eight weeks.

Indirect DST was performed on all positive isolates using absolute concentration measurement (MIC) on LJ media to determine susceptibility to isoniazid (0.2 and 1 ug/ml); rifampicin (32 and 64 ug/ml); ethambutol (2.8 and 4 ug/ml); and streptomycin (8 and 16 ug/ml). [16] Time to detection of growth and contamination rates were recorded for each type of culture medium.

The second sputum aliquot underwent MODS testing in accordance with published standard operating procedures. (28) All MODS test results were interpreted without knowledge of the results of the reference standard. Briefly, the sample was decontaminated using a sodium hydroxide-sodium citrate-NaCl solution and inoculated into Middlebrook 7H9 liquid broth containing OADC and PANTA. 900 µl of this sample-broth mixture was aliquoted into each of four well columns in a 24 microtitre well plate; for each patient sample, the first two wells were drug-free, the third well contained 100 µl isoniazid at 0.4 ug/ml concentration, and the fourth well contained rifampicin at 1 ug/ml. Plates were incubated at 37°C. MODS cultures were examined using an inverted light microscope at 340 magnification daily from day 4 through day 21, and thereafter weekly through 40 days incubation. Positive MODS cultures were defined by the presence of characteristic cord formation at time of detection of growth.

During the study period, the lab transitioned from standard MODS to use of the TB MODS Kit™ (Hardy Diagnostics, Santa Maria, CA USA). Briefly, specimens were decontaminated as for standard MODS and inoculated into commercially prepared vials of Middlebrook 7H9 liquid broth containing OADC to which 100 µl of PANTA was added prior to sample inoculation. Direct patient samples were inoculated into two drug-free plate wells, one plate well containing isoniazid at 0.4 ug/ml concentration, and one plate well containing rifampicin at 1 ug/ml, with examination performed as with standard MODS. Validation reports from the manufacturer document a diagnostic accuracy similar to or greater than standard MODS [17].

### Statistical Analysis

We calculated proportions with exact binomial 95% confidence intervals (CI) for the primary analyses of sensitivity, specificity, positive predictive value, and negative predictive value. A positive reference for *M. tuberculosis* detection was defined as a positive result on either LJ or MGIT culture. Time to positivity (TTP) was defined as the time from inoculation of the specimen in the laboratory to report of test positivity. In order to generate conservative estimates of diagnostic accuracy, we included indeterminate or contaminated MODS results in the denominator for calculation of sensitivity if they occurred in individuals with culture-positive TB, while indeterminate or contaminated MODS results were excluded from analysis for calculation of specificity. For categorical variables, we compared proportions using chi-square tests; for continuous variables, we compared medians using the Wilcoxon rank-sum test. TTP was assessed using survival analysis, with treatment arms compared using the log-rank test. All *P* values were two-sided with alpha = 0.05 as the significance level. Data analysis was performed using Stata 12.1 (Stata Corporation, College Station, Texas).

## Results

### Patients and Samples

**Figure 1:**
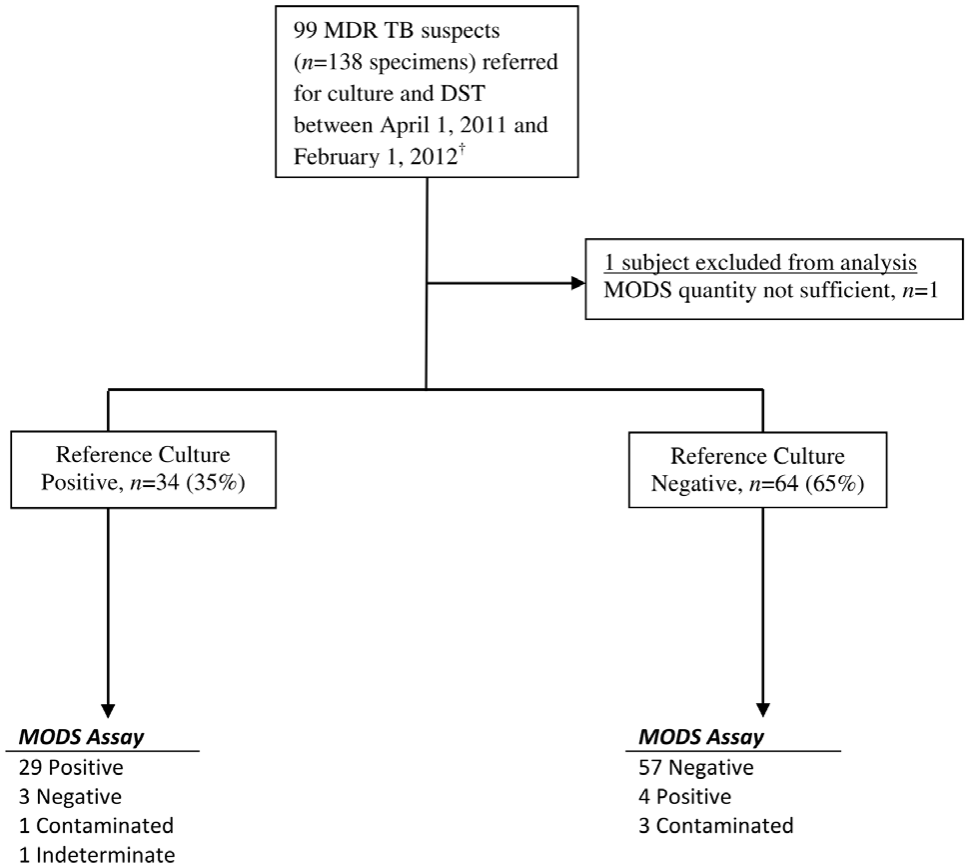
Study Flow Diagram. *Definition of abbreviation*: MDR TB = multidrug resistant tuberculosis; MODS = microscopic-observation drug-susceptibility. ^†^Six additional previously cultured isolates of known MDR status were included for analysis of drug susceptibility testing only and are not included here.

One hundred thirty-eight sputum samples were collected from 99 drug-resistant TB suspects, of whom 40% were female and the median age was 37 years (interquartile range [IQR]: 27–44). Retreatment category was available for 86 (87%) patients; 12/86 (14%) had no prior TB history but were contacts of known or suspected MDR-TB cases (Table 1). Among persons with known HIV status, 39/59 (66%) were HIV-infected. One patient was excluded from further analysis due to insufficient sample quantity for MODS (Figure 1).

**Table 1:** Characteristics of the Study Population.

Characteristic	Total, *n* = 99
**Age, years, median (IQR)**	37 (27–44)
**Male, %**	60
**HIV-infected,** * **%**	66
** CD4+ T-cell count, median cells/uL (IQR)** †	160 (84–285)
**Site of referral, %**	
** Outpatient clinic**	73
** Inpatient ward**	27
**Reason for referral, %** ‡	
** Default**	6
** Relapse**	20
** Treatment failure, Category I**	20
** Treatment failure, Category II**	13
** Contact with known/suspected MDR case**	12
** Other retreatment**	12
** Unknown/Not recorded**	17
**TB treatment (any) at time of sample collection, %**	80
**Sputum AFB smear result, %**	
** AFB smear-negative**	70
** AFB smear-positive**	30
**Number of samples collected, %**	
** One**	82
** ≥ Two**	18
**MODS testing format, %**	
** Standard MODS**	64
** TB MODS Kit™** §	36

Values are percentages unless otherwise stated. All categories are mutually exclusive. The denominator for each characteristic excludes missing or unknown values unless otherwise stated.

Definition of abbreviations: MODS, microscopic-observation drug-susceptibility assay.

* Among persons with known HIV status (*n* = 59/99 (60%)).

† Available for *n* = 27/59 (46%) HIV-infected persons.

‡ Retreatment categories were defined according to World Health Organization criteria; [1] smear- or culture-positivity at the fifth month or later was defined as treatment failure, stratified according to Category I or Category II treatment at the time failure occurred.

§ Hardy Diagnostics, Santa Maria, CA USA.

### *M. tuberculosis* Detection

*M. tuberculosis* was identified in 34/98 (35%) clinical samples from either solid or liquid culture. Of these, 19/34 (56%) were MDR, 9/34 (27%) were drug-susceptible, 1/34 (3%) was rifampin-monoresistant, and 5/34 (15%) identified *M. tuberculosis* but were contaminated prior to finalization of DST. Eighteen percent (n = 6/34) of cases were smear-negative. Overall MODS sensitivity for *M. tuberculosis* detection was 85% (95% CI, 69–95%) and specificity was 93% (95% CI, 84–98%) when compared with the reference standard of solid or manual liquid culture (Table 2). The negative predictive value for excluding TB among drug-resistant TB suspects was 92% (95% CI, 82–97%). In an analysis stratified by HIV status, neither sensitivity (85% for HIV-positive, 86% for HIV-negative; p = 0.53) nor specificity (92% for HIV-positive, 100% for HIV-negative; p = 0.31) demonstrated statistically significant differences.

**Table 2:** Comparison of the Microscopic-observation Drug-Susceptibility (MODS) Assay with Reference Standard Culture for Detection of *M. tuberculosis*.

MODS Assay	Reference Standard Culture
No. of samples positive for *M. tuberculosis* by reference standard method (%)	34 (35)
All directly inoculated samples (n = 98)	
Sensitivity, % (95% CI)	85 (69–95)
Specificity, % (95% CI)	93 (84–98)
Positive predictive value, % (95% CI)	88 (72–97)
Negative predictive value, % (95% CI)	92 (82–97)
HIV-positive (n = 39)*	
Sensitivity, % (95% CI)	85 (55–98)
Specificity, % (95% CI)	92 (73–99)
Positive predictive value, % (95% CI)	85 (55–98)
Negative predictive value, % (95% CI)	92 (73–99)
HIV-negative (n = 20)*	
Sensitivity, % (95% CI)	86 (42–100)
Specificity, % (95% CI)	100 (74–100)
Positive predictive value, % (95% CI)	100 (54–100)
Negative predictive value, % (95% CI)	93 (64–100)

* Among persons with known HIV status (*n* = 59/98 (60%)).

Among the five TB cases not detected by the MODS assay (i.e., “false-negatives”), one was MODS indeterminate with mixed *M. tuberculosis*/nontuberculous mycobacteria (NTM) noted on manual MGIT culture, one was MODS contaminated, and two were smear-negative with growth detected by MGIT following prolonged incubation (>21 days). Among four reference standard-negative, MODS-positive samples (i.e., “false-positives”), two were MODS-positive following prolonged incubation (>21 days), and one was shown to be multidrug resistant upon manual MGIT culture of a separately collected patient specimen.

Initial contamination (including specimens that were later successfully decontaminated) was similar for the MODS assay (*n* = 9/138 (6.5%) specimens), manual MGIT (*n* = 15/138 (10.9%), and LJ culture (*n* = 8/138 (5.8%); p = 0.81). Although power was limited, no difference in contamination was noted with use of the TB MODS Kit™ (6%) versus standard MODS (6%; p = 0.43 for difference).

### Drug Susceptibility Testing

Of 29 specimens positive by both MODS and reference standard solid LJ or manual MGIT culture, two were absolute concentration method-indeterminate due to contamination of sub-culture. Therefore, 27 directly inoculated patient specimens and six previously cultured specimens had concurrent MODS isoniazid and rifampin wells for comparison with the absolute concentration method. Among directly inoculated samples, resistance to isoniazid was detected in 18/27 (67%), to rifampin in 18/27 (67%), and to both isoniazid and rifampin (i.e., MDR-TB) in 17/27 (63%) by the reference standard. The MODS assay successfully detected isoniazid and rifampin resistance among all six previously cultured (i.e., indirectly inoculated) specimens. Overall sensitivity of the MODS assay for detection of resistance to isoniazid, rifampin, and MDR-TB was 88% (95% CI, 68–97%), 96% (95% CI, 79–100%), and 91% (95% CI, 72–99%), respectively; specificity was 89% (95% CI, 52–100%), 89% (95% CI, 52–100%), and 90% (95% CI, 56–100%), respectively (Table 3).

**Table 3:** Drug-Susceptibility Test Results from the MODS Assay.

	Isoniazid	Rifampin	Isoniazid+Rifampin (multidrug resistance)
No. of samples*	33	33	33
No. resistant (prevalence)†	18/27 (67%)	18/27 (67%)	17/27 (63%)
Sensitivity, % (95% CI)	88 (68–97)	96 (79–100)	91 (72–99)
Specificity, % (95% CI)	89 (52–100)	89 (52–100)	90 (56–100)
Positive predictive value, % (95% CI)	96 (77–100)	96 (79–100)	96 (77–100)
Negative predictive value, % (95% CI)	73 (39–94)	89 (52–100)	82 (48–98)

* Analysis limited to samples with positive microscopic-observation drug-susceptibility and reference standard culture.

† Among directly inoculated patient specimens.

### Time to Positivity for Detection of *M. tuberculosis* and Drug Resistance

**Figure 2:**
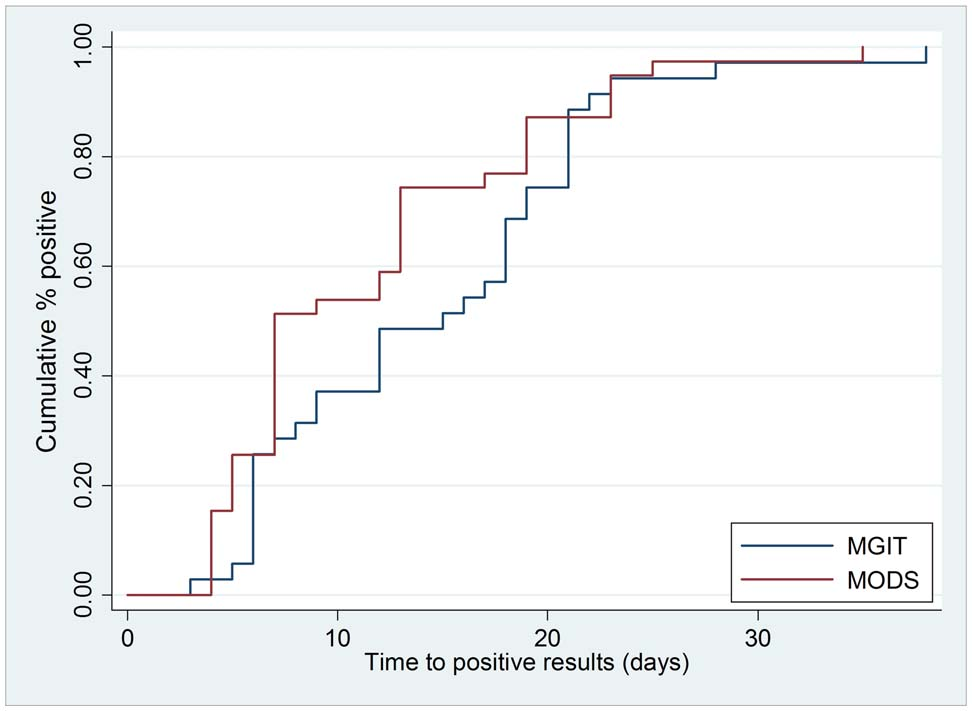
Kaplan-Meier Curves of Time to *M. tuberculosis* Detection. Time to positivity for *Mycobacterium tuberculosis* detection for microscopic-observation drug-susceptibility (MODS) and reference standard culture (manual mycobacterial growth indicator tube (MGIT)). Median time to positivity was significantly shorter for MODS than for manual MGIT (MODS 7 days [IQR 7–15 days] vs. MGIT 12 days [IQR 6–16 days]; p<0.001).

Overall, the median time to culture positivity was significantly shorter for MODS than for the manual MGIT liquid or LJ cultures (MODS 7 days [IQR 7–15 days] vs. MGIT 12 days [IQR 6–16 days] vs. LJ 28 days [IQR 21–35 days]; p<0.001) (Figure 2). Median time to positivity for MODS MDR-TB diagnosis (7 days [IQR 7–15 days]) was significantly shorter than that for the absolute concentration method (71 days [IQR 51–75 days]; p<0.001).

## Discussion

This operational study evaluated the performance of the MODS assay among drug-resistant TB suspects living in a high HIV-prevalence setting. MODS detected *M. tuberculosis* and associated drug resistance with high sensitivity and shorter time to positivity compared with reference standard culture and DST methods. Given the expanding global prevalence of MDR-TB/HIV and continued need for an affordable, accurate, and rapid point-of-care test, these findings have implications for other limited-resource settings [18], [19].

Zimbabwe has among the highest TB incidence per capita (603/100,000) in the world, [1] with approximately 70% of active TB cases occurring among individuals co-infected with HIV. [20] Although HIV prevalence has declined since the 1990s, 16% of the adult population remains HIV-infected. [21] The World Health Organization estimates the prevalence of MDR-TB in Zimbabwe among patients with a prior history of TB treatment to be 8.3% (95% CI, 3–20%), [12] though these data were collected in 1995 and the current extent of drug resistant-TB in the country is unknown. That prevalence of MDR-TB has increased in the country in the context of severe economic destabilization, challenges to tuberculosis control, and population displacement has been suggested, [22] though supporting evidence is thus far lacking.

Expanded capacity to perform DST in high burden settings is a critical need. In countries where mycobacterial culture is not routinely utilized, failure of one or more regimens of TB drugs is typically a prerequisite for referral for DST. Thus, 12 or more months often elapse from clinical presentation to MDR-TB confirmation. Given high early mortality [23] and the potential for ongoing transmission, [24] expedited diagnosis and early institution of effective therapy is life-saving and a critical public health mandate. Although debate exists as to best scale-up option for DST in resource limited settings, [25] the high accuracy, low cost, ability to discern both isoniazid and rifampicin resistance, relative ease of operational implementation and short turnaround time should make MODS a strong consideration.

Consistent with the single other study assessing MODS diagnostic accuracy among TB suspects in a high-HIV prevalence region, [10] we found somewhat lower sensitivity for *M. tuberculosis* detection than that reported from other settings. [26] In our study, most false-negative specimens either required prolonged incubation prior to positivity or were considered false-negative due to MODS contamination. Although culture contamination was not dissimilar to that reported by other investigators, [7], [26] contamination of liquid mycobacterial cultures is a known challenge for routine laboratories in sub-Saharan Africa. [27] Sensitivity for detection of isoniazid and rifampicin resistance was similar to previously reported studies, [7] though negative predictive value was lower due to the high prevalence of drug resistance noted among MDR-TB suspects in this high burden setting; negative predictive value would be marginally higher when testing new patients without history of prior TB treatment (i.e., those at lower risk for drug resistance). Further, the sensitivity for detection of isoniazid resistance could be increased through use of a lower MIC (0.1ug/ml) cutpoint in MODS. [28] Of note, diagnostic accuracy in studies of drug susceptibility testing is dependent upon choice of denominator for analysis. With a denominator including patients with reference standard culture-positive disease (as opposed to a denominator including specimens culture-positive by both index test and reference standard), MODS sensitivity and negative predictive value for detection of drug resistance would be marginally lower.

Although upfront costs are higher relative to standard, noncommercial MODS (approximately $5.00 per test versus $1.48 for standard MODS), [26] it has been anticipated that use of the TB MODS Kit™ (Hardy Diagnostics, Santa Maria, CA USA) will improve biologic security, attention to published standard operating procedures, and adherence to quality assurance systems. While validation data reported by the manufacturer are excellent, [17] diagnostic accuracy studies by independent investigators are necessary and are underway. In the current study, no meaningful difference in diagnostic accuracy was noted between the “in-house” noncommercial MODS assay and the commercial kit, though power for this determination was limited.

A strength of our study is its operational, real-world nature. However, threats to internal or external validity include the following. First, as in many settings, routine DST of retreatment TB cases in Harare, Zimbabwe is codified in policy though not yet standard practice due to resource limitations, and our sample must be regarded as one of convenience. Further, we were unable to ensure standardization of specimen collection and processing for routinely collected samples, and cannot rule out the possibility that some false-negative results may have been due to suboptimal quality in these areas; similarly, data was incomplete and collected retrospectively for some individuals. Second, power to detect meaningful differences in our HIV-stratified analysis was limited. However, that our point estimates are similar to a recent adequately powered study from a setting of similar HIV prevalence [10] lends confidence to our results. Third, we were unable to undertake comprehensive microbiologic, molecular, and epidemiologic investigation into discordant cases. Our use of a reference standard including both solid and liquid culture methods provided a rigorous definition of true positive results. Last, diagnostic accuracy is a surrogate for patient-important outcomes such as time to treatment initiation and mortality.

In conclusion, MODS detected *M. tuberculosis* and *M. tuberculosis* drug resistance with high sensitivity and more rapid time to positivity compared with standard culture and DST methods. Further, no detectable differences in diagnostic accuracy were noted for HIV-infected patients. Prompt treatment of patients with MDR-TB and screening of their contacts will be essential to prevent further spread of drug-resistant *M. tuberculosis*; that this will occur within the context of continued socioeconomic stabilization and improved health service delivery is hoped for and anticipated. Studies focused on patient-important outcomes, along with valid sampling methods to generate accurate estimates of the prevalence of MDR-TB in modern-era Zimbabwe, are urgently needed.
